# The Saccharide Mapping and Biological Activity Analysis of *Auricularia heimuer* Polysaccharides from Different Origins In Vitro

**DOI:** 10.3390/foods15213816

**Published:** 2026-10-26

**Authors:** Miao Ding, Zixuan Wang, Hongxiang Liu, Yingdi Dai, Yang Liu, Qi Wang

**Affiliations:** Engineering Research Center of Chinese Ministry of Education for Edible and Medicinal Fungi, College of Plant Protection, College of Mycology, Jilin Agricultural University, Changchun 130118, China; miaoding@mails.jlau.edu.cn (M.D.); 13948530370@163.com (Z.W.); hongxiangliu@jlau.edu.cn (H.L.); daiyingdi@mails.jlau.edu.cn (Y.D.)

**Keywords:** *Auricularia heimuer*, polysaccharide, physicochemical property, biological activity

## Abstract

*Auricularia heimuer* (*A. heimuer*) polysaccharides (AHPs) are the main bioactive components of edible and therapeutic fungi. AHPs have considerable potential for use in healthy meals and supplements; however, because of their large molecular weight and structural complexity, quality control remains difficult. A total of 32 *A. heimuer* samples were collected from various geographic locations. High-performance liquid chromatography (HPLC), high-performance gel permeation chromatography (HPGPC-ELSD), Fourier transform infrared spectroscopy (FT-IR), and high-performance thin-layer chromatography (HPTLC) were used to characterize the physicochemical properties and saccharide profiles of AHPs. The findings showed that Man and Glc constituted the majority of AHPs, with molecular weights varying between 3.6 and 6820 kDa. All samples demonstrated in vitro antioxidant, lipid-lowering, and hypoglycemic properties in a concentration-dependent manner. Critical structure–activity relationships were identified through correlation analysis: uronic acid content (expressed as galacturonic acid equivalents), Glc, and Man were linked to antioxidant activity, while Man, Xyl, specific molecular weight ranges, and distinctive FT-IR absorption peaks (1731, 1376, 1245, and 1656 cm^−1^) were linked to hypoglycemic and lipid-lowering effects. This research provides support for quality grading, functional development, and industrial standardization of *A. heimuer* production areas.

## 1. Introduction

*Auricularia heimuer* (*A. heimuer*), also known as tree ear, cloud fungus, or wood ear, is named for the ear-like shape of its fruiting body [1]. It has a long history of use as an edible and medicinal fungus in China [2]. Taxonomically, *A. heimuer* belongs to the phylum Basidiomycota, class Agaricomycetes, order Auriculariales, family Auriculariaceae, and genus *Auricularia* [3]. The edible fruiting body is ear-shaped, translucent, and elastic, with a smooth concave surface and a densely hairy underside [4]. *A. heimuer* is distributed across more than 20 provinces and autonomous regions of China, with Yunnan, Sichuan, Henan, Hubei, Shaanxi, Jilin, and Heilongjiang among the major production areas [5]. In 2023, the China Edible Fungi Association reported that the annual production of *A. heimuer* reached 7.145 million tons, with a reported production value of 396.56 billion yuan, indicating its substantial production scale and economic importance. Following *Lentinula edodes*, *A. heimuer* is one of the major edible mushroom species cultivated in China.

The focus on *A. heimuer* has expanded beyond flavor and yield to nutritional roles as public health awareness has increased. *A. heimuer* contains polysaccharides (AHPs), proteins, dietary fiber, and trace elements. AHPs, the primary active constituents of *A. heimuer*, exhibit anticancer, hypoglycemic, hypolipidemic, immunomodulatory, and antioxidant properties, making them a major area of study in natural product research [6]. However, it is challenging to precisely assess and differentiate the quality of *A. heimuer* because of the dearth of systematic comparative studies on AHPs from various origins. In contrast, systematic studies on polysaccharides from various edible and medicinal fungi, including *Ganoderma lucidum*, *Flammulina velutipes*, and *Hericium erinaceus*, have been conducted [7,8,9].

In this study, 32 *A. heimuer* samples from various origins were collected, and a quality evaluation system was constructed by measuring the total sugar, protein, and uronic acid content (expressed as galacturonic acid equivalents), as well as the molecular weight distribution, monosaccharide composition, and in vitro activity. This study aimed to provide a theoretical foundation and statistical backing for the scientific grading, functional product development, and industrial standardization of *A. heimuer* quality.

## 2. Materials and Methods

### 2.1. Samples and Reagents

The sources of the 32 *A. heimuer* samples collected in this study are presented in Table 1. Subsequent to sample collection, the specimens were cleaned, desiccated, pulverized, and sieved through an 80-mesh sieve for future utilization. Anhydrous glucose, galacturonic acid, bovine serum albumin (BSA), dextran standard, and 3,5-dinitrosalicylic acid (DNS) reagent were obtained from Solarbio Technology Co., Ltd. (Beijing, China). Analytically pure anhydrous ethanol, phenol, concentrated sulfuric acid, m-hydroxybiphenyl, sodium tetraborate, sodium chloride, potassium bromide (chromatographic grade), aniline, diphenylamine, 85% phosphoric acid, and acetone were acquired from Sinopharm Chemical Reagents Co., Ltd. (Shanghai, China). DPPH and hydroxyl radical scavenging ability test kits were purchased from Nanjing Jiancheng Bioengineering Research Institute Co., Ltd. (Nanjing, China). The superoxide anion scavenging capability test kit was purchased from Box Biotechnology Co., Ltd. (Beijing, China). The BCA Protein Quantification Kit was purchased from Elabscience Biotechnology Co., Ltd. (Wuhan, China). Lauric acid-4-nitrophenyl ester and phthalate were purchased from Aladdin Biochemical Technology Co., Ltd. (Shanghai, China). The p-Nitrophenyl butyrate was obtained from Sigma-Aldrich (St. Louis, MO, USA). Monosaccharide standards, including fucose (Fuc), arabinose (Ara), galactose (Gal), glucose (Glc), rhamnose (Rha), xylose (Xyl), mannose (Man), glucuronic acid (GlcA), β-mannosidase, β-glucosidase, pectinase, cellulase, pancreatic lipase, cholesterol esterase, starch, and α-amylase, were acquired from Shanghai Yuanye Biotechnology Co., Ltd. (Shanghai, China). Maltose (SJ-MN1316), maltotriose (SJ-MN2680), maltotetraose (SJ-MN1309), and maltopentaose (SJ-MN1226) were obtained from Shandong Sparkjade Biotechnology Co., Ltd. (Jinan, China).

### 2.2. Polysaccharide Extraction

AHPs were extracted with hot water at a solid-to-liquid ratio of 1:30 (g/mL) for 3 h at 95 °C. The supernatant was concentrated to approximately one-tenth of its original volume at 50 °C using a rotary evaporator under reduced pressure. Three volumes of ethanol were then added, and the mixture was kept at 4 °C overnight. The mixture was centrifuged at 6000 rpm for 15 min to collect the crude polysaccharide precipitate. The residual ethanol was allowed to evaporate, and the precipitate was redissolved in reverse osmosis (RO) water. The solution was deproteinized using the Sevag method, followed by dialysis against RO water using a dialysis membrane (MWCO: 3500 Da; JielePu, Hunan Yibo Biotech, Changsha, China) for 48 h. After dialysis, the aqueous solution was filtered using a vacuum filter (Guangzhou Jet Bio-Filtration Co., Ltd., Guangzhou, China) and subsequently freeze-dried to obtain AHPs [10].

### 2.3. Chemical Composition Determination

The total sugar level was quantified using the phenol-sulfuric acid technique with a glucose standard [11]. Optical density values at 490 nm were recorded following the amalgamation of glucose solutions (0–0.1 mg/mL) and AHP solutions (0.1 mg/mL) with 6% phenol and concentrated sulfuric acid. A standard curve was established with glucose concentration on the x-axis and optical density (OD) value on the y-axis, and the regression equation was used to determine the total sugar content of the AHPs.

Protein content was determined using a BCA kit with a bovine serum albumin standard [12]. BSA standard solutions (0–2000 μg/mL) and AHP solutions (0.1 mg/mL) from various sources were prepared. The BCA working solution was prepared according to the manufacturer’s guidelines, and the optical density (OD) was recorded at 595 nm. A standard curve was constructed using BSA concentrations and OD values to determine the protein content of the AHPs.

The uronic acid content was determined using the m-hydroxybiphenyl method with a galacturonic acid standard [13]. Standard solutions of galacturonic acid (0–0.1 mg/mL) and AHP solutions (0.1 mg/mL) were prepared. Subsequently, 3 mL of sodium tetraborate-sulfuric acid solution was added, and the mixture was boiled for 5 min. The solution was then cooled to room temperature (RT). Following the addition of the m-hydroxybiphenyl solution and a 30 min incubation period, absorbance was measured at 520 nm. A standard curve was constructed using galacturonic acid concentration to determine the uronic acid contentin the AHPs.

### 2.4. Monosaccharide Composition Determination

The monosaccharide content of AHPs from various sources has been analyzed using HPLC [14]. The chromatographic parameters employed were a Shimadzu LC-20AT, an SPD-20A ultraviolet–visible detector, and a COSMOSIL 5C18-PAQ column measuring 4.6 mm ID × 250 mm at 35 °C, with a 10 μL injection volume and a flow rate of 1 mL/min. The sugar constituents of the AHPs from various sources were ascertained by comparing the retention duration and peak area with monosaccharide standards (Fuc, Ara, Gal, Glc, Rha, Xyl, Man, GalA, and GlcA).

### 2.5. Molecular Weight Determination

The molecular weights of AHPs from different origins were determined using HPGPC-ELSD [15]. The stipulations were as follows: 0.2 mol/L sodium chloride aqueous solution served as the mobile phase, with elution conducted at a flow rate of 0.5 mL/min. A standard curve was constructed with the sample retention time on the x-axis and the logarithm of the dextran relative molecular weight on the y-axis, from which the molecular weights of the samples were derived.

### 2.6. FT-IR Analysis

Chromatographic-grade KBr (190 mg) and AHPs (1.9 mg) were dried at 110 °C until a consistent weight was achieved. The amalgamation was pulverized, compressed into sheets using an agate mortar, and examined by FT-IR (4000–500 cm^−1^) [16].

### 2.7. HPTLC Analysis

AHP samples (5 mg/mL, 1 mL) were separately mixed with 0.5 mL of β-mannosidase (20 U/mL), β-glucosidase (10 U/mL), pectinase (20 U/mL), cellulase (10 U/mL), or α-amylase (20 U/mL). The mixtures were then incubated at 37 °C for 12 h. Subsequent to enzymatic hydrolysis, the solution was heated at 80 °C for 30 min to inactivate the enzymes. Denatured proteins were eliminated by centrifugation, and the supernatants were evaporated to yield the hydrolysis products. Standard solutions of glucose, maltose, maltotriose, maltotetraose, and maltopentaose were prepared in 60% ethanol. The developing solvent consisted of n-butanol, isopropanol, glacial acetic acid, and water at a volume ratio of 7:5:1:3. The chromogenic reagent comprised 4 g of aniline, 4 mL of diphenylamine, and 20 mL of 85% phosphoric acid dissolved in 200 mL of acetone. Samples and standards were applied to high-performance thin-layer chromatography (HPTLC) plates in 0.5-cm-wide strips, spaced 1 cm apart, with application starting 1 cm from the bottom edge. Subsequent to drying, the plate was developed in a saturated tank for 30 min, dried again, sprayed with a color developer, and subjected to heating at 85 °C for 10 min [17].

### 2.8. Antioxidant Activity Analysis

The scavenging abilities of the extracts against DPPH radicals, hydroxyl radicals, and superoxide anions were assessed following the manufacturer’s guidelines. Solutions of AHPs (0.2, 0.4, 0.8, and 1 mg/mL) from various sources were prepared and reacted with the appropriate reagents in the kits. Absorbance values were recorded at 517, 550, and 530 nm, and the free radical scavenging activity was determined [18].

### 2.9. Lipid-Lowering and Hypoglycemic Potentials’ Analysis

The inhibitory action of pancreatic lipase was assessed using lauric acid-4-nitrophenyl ester as the substrate [19]. Solutions of AHPs (0.2, 0.4, 0.8, and 1 mg/mL) were introduced into centrifuge tubes containing 60 μL of pancreatic lipase, 60 μL of PBS, 20 μL of AHP solution, and 80 μL of the substrate solution. After 30 min at 35 °C, the optical density was measured at 405 nm. A dose–effect curve was constructed by plotting the polysaccharide concentration against the inhibition rate to quantify the pancreatic lipase inhibition of AHPs.

Cholesterol esterase inhibitory activity was identified using a p-nitrophenyl butyrate substrate [20]. Solutions of AHPs (0.2, 0.4, 0.8, and 1 mg/mL) comprised cholesterol esterase, polysaccharide solution, PBS buffer (1 mL), and substrate solution (20 μL), and were introduced into centrifuge tubes. Following a 30 min incubation at 25 °C, the optical density was assessed at 405 nm. A standard curve was constructed to depict the polysaccharide content against the inhibition rate to determine the cholesterol esterase inhibition of AHPs.

The cholesterol adsorption capability under simulated in vivo conditions was quantified by colorimetry using phthalaldehyde [21]. AHPs (20 mg) were incorporated into 1 mL of egg yolk solution at pH 2.0 and 7.0. After incubation at 37 °C for 2 h and subsequent centrifugation, the supernatant and cholesterol standards (0.2, 0.4, 0.8, and 1 mg/mL) were treated with phthalaldehyde and an acid combination. The absorbance was recorded at 550 nm, and the cholesterol adsorption capacity was determined using a regression equation derived from the standard curve.

α-Amylase inhibitory activity was assessed by DNS colorimetry using starch [22]. AHP solutions (0.2, 0.4, 0.8, and 1 mg/mL) were introduced into centrifuge tubes along with enzyme, polysaccharide, starch, and DNS solutions. The optical density was measured at 540 nm after boiling. A standard curve was constructed to depict the polysaccharide content against the inhibition rate to determine the α-amylase inhibition rate of the AHPs.

### 2.10. Pearson Correlation Analysis

Pearson correlation analysis was performed using Origin 2025 to evaluate the relationships between the physicochemical characteristics and biological activities of AHPs obtained from 32 different geographical origins. The physicochemical variables included total sugar content, protein content, and uronic acid content (expressed as galacturonic acid equivalents), monosaccharide composition (mannose, glucuronic acid, glucose, galactose, xylose, and fucose), molecular weight distribution parameters (Peak 1–Peak 5), and characteristic FT-IR absorption bands. The biological activity variables included the IC_50_ values for DPPH, hydroxyl, and superoxide radical-scavenging activities, α-amylase inhibition, pancreatic lipase inhibition, and cholesterol esterase inhibition, as well as cholesterol adsorption capacity. Pearson correlation coefficients (r) and corresponding *p*-values were calculated to assess the strength and direction of the linear associations between physicochemical characteristics and biological activities. Correlations with *p* < 0.05 were considered statistically significant, whereas correlations with *p* < 0.01 were considered highly significant. The resulting correlation matrix was visualized as a heatmap.

### 2.11. Statistical Analysis

Experiments were conducted in three parallel replicates, and the findings were presented as the mean ± standard deviation (mean ± SD). Statistical analysis was conducted using SPSS Statistics 26.0 software (IBM Corp., Armonk, NY, USA), employing one-way analysis of variance (ANOVA), with *p* < 0.05 denoting significance and *p* < 0.01 indicating extreme significance.

## 3. Results and Discussion

### 3.1. Chemical Composition of AHPs from Different Origins

Table 2 presents the total sugar, protein, and uronic acid content of 32 AHP samples from different locations. The total sugar content ranged from 56.89% to 79.90%, whereas the protein content was below 10%, indicating that all samples contained relatively high levels of sugars. The total uronic acid content, expressed as galacturonic acid equivalents, ranged from 10.72% to 20.34%. Uronic acids are important components of acidic polysaccharides and may influence their physicochemical properties, including water solubility and negative charge density, as well as their antioxidant and immunomodulatory activities. Sample No. 7 exhibited the highest uronic acid content, whereas Sample No. 16 had the lowest value. The relatively low uronic acid content of Sample No. 16 may indicate a lower degree of acidic character than that of the other samples, although further structural characterization would be required to confirm its polysaccharide features [23].

### 3.2. Monosaccharide Composition of AHPs from Different Origins

**Figure 1:**
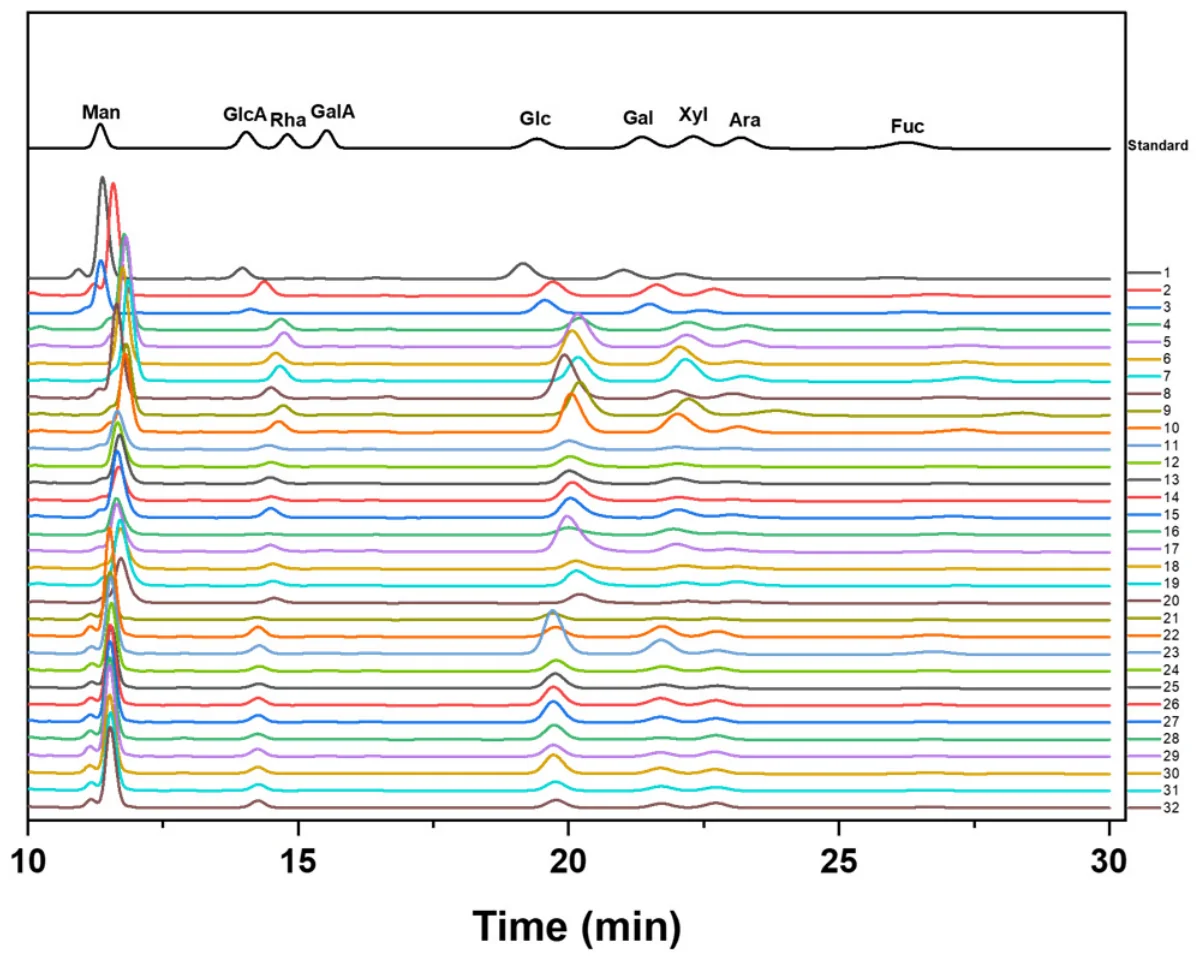
The HPLC chromatogram of monosaccharide standards and AHPs from different origins.

The monosaccharide composition characteristics of AHPs from 32 origins were shown in Table 3 and Figure 1, and were mainly composed of six monosaccharides: Man, GlcA, Glc, Gal, Xyl and Fuc. Although the composition of monosaccharides from different origins was similar, the proportion of monosaccharides from each origin was significantly different, which was consistent with the results of a saffron polysaccharide study by Zhang et al. [18]. Man and Glc are the major monosaccharides in AHPs, collectively accounting for 50–80% of their total monosaccharide composition. The Man content varied from 32.3% to 65.6%, with the maximum recorded at No. 21 (65.6%) and the minimum at No. 9. Glc constituted 8.8–42.6%, with No. 17 exhibiting a much higher value, while No. 22 recorded the lowest at 8.8%. Fuc had a non-uniform distribution, with 11 origins undetected and the remaining 21 origins comprising 1.9–5.5%.

### 3.3. Molecular Weights of AHPs from Different Origins

The molecular weight distribution of AHPs from 32 different origins was determined by high-performance gel permeation chromatography (HPGPC). As shown in Table 4, five chromatographic peaks (Peak 1–Peak 5) were identified in the molecular weight profiles of the AHP samples [1]. The lowest molecular weight of Peak 1 was observed in sample 32 (401 kDa). Peak 2 showed a molecular weight range of 12.2–4791.1 kDa, with the highest value detected in sample 20 (4791.1 kDa). Peak 3 ranged from 4.1 to 2850.4 kDa, with the highest molecular weight observed in sample 14 (2850.4 kDa). Peak 4 represented a relatively low-molecular-weight fraction, ranging from 3.6 to 13.1 kDa. Peak 5 was detected in only a subset of AHP samples and exhibited molecular weights ranging from 3.6 to 4.8 kDa, with the highest value observed in sample 30 (4.8 kDa).

### 3.4. FT-IR of AHPs from Different Origins

**Figure 2:**
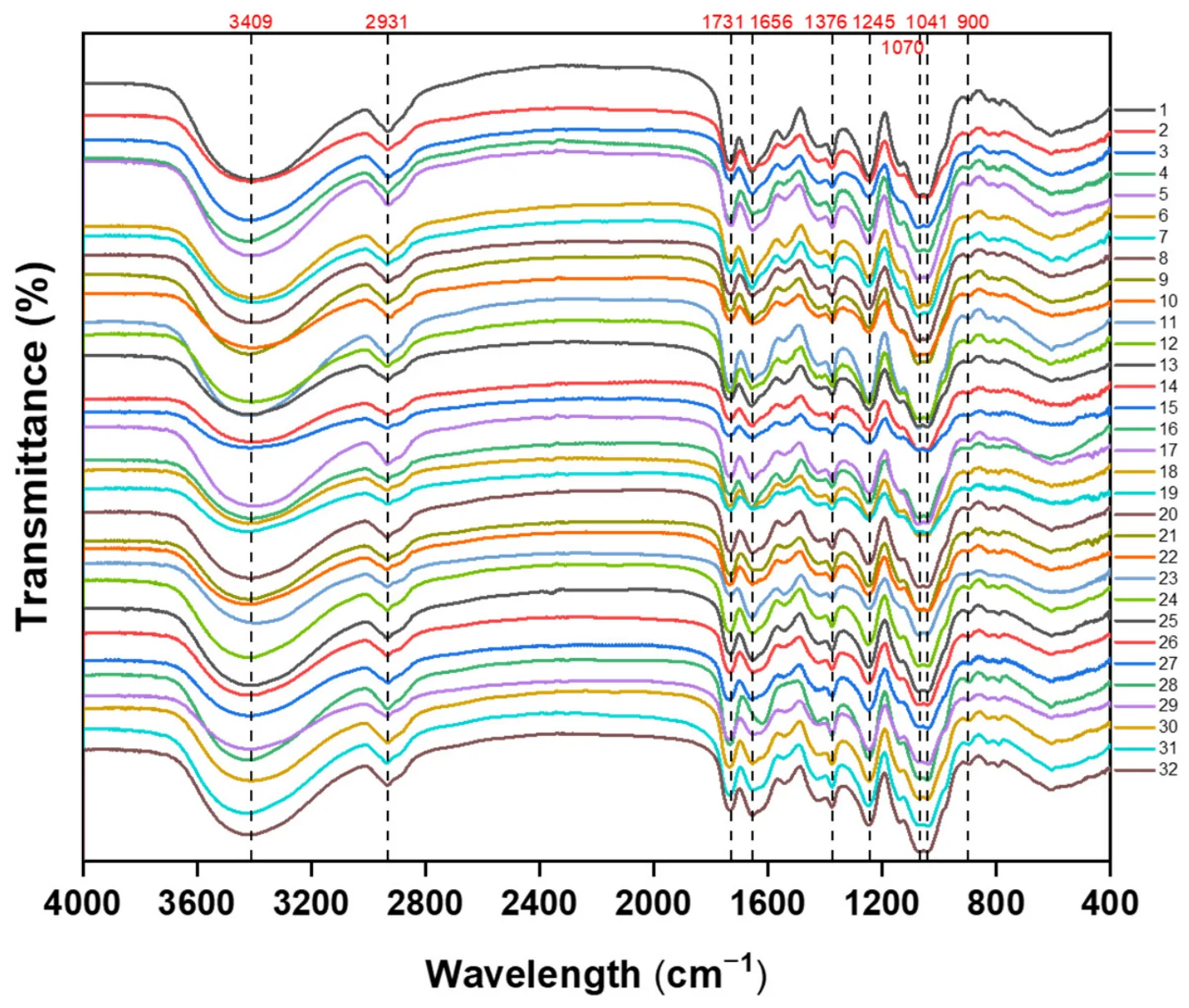
FT-IR spectra of AHPs from different origins.

Figure 2 showed similar peak profiles and positions among AHPs from different origins, although differences in absorption intensity were observed, indicating that these samples shared similar chemical structures but varied in the abundance of certain functional groups [24]. The broad absorption band at 3409 cm^−1^ was attributed to O–H stretching vibrations associated with hydroxyl groups, which are characteristic functional groups of polysaccharides and contribute to hydrogen bonding interactions [25]. The absorption peaks at 2931 and 1376 cm^−1^ were assigned to C–H stretching and bending vibrations of CH, CH_2_, and CH_3_ groups, reflecting the presence of sugar ring structures [26]. The absorption band at 1731 cm^−1^ was attributed to C=O stretching vibrations of esterified carboxyl groups, potentially associated with the methyl esterification of galacturonic acid residues in AHPs [27,28]. The absorption band at 1656 cm^−1^ was associated with bound water and/or amide group vibrations, indicating possible interactions between polysaccharide chains and minor non-polysaccharide components [29]. The peak at 1245 cm^−1^ was related to C–O stretching vibrations [30]. The characteristic absorption bands at 1070 and 1041 cm^−1^ were attributed to C–O–C and C–O stretching vibrations of pyranose ring structures, which are typical structural features of fungal polysaccharides [31]. Moreover, the absorption band around 900 cm^−1^ suggested the presence of β-glycosidic linkages [32]. Collectively, these spectral characteristics suggested that AHPs from different origins shared similar polysaccharide structural features, including hydroxyl groups, uronic acid residues, pyranose ring structures, and β-glycosidic linkages. The variations in peak intensities may be associated with differences in the relative abundance or local chemical environments of these functional groups.

### 3.5. HPTLC of AHPs from Different Origins

The HPTLC profiles of enzymatically hydrolyzed polysaccharides are shown in Figure S1 and Table 5. Figure S1A showed no obvious bands for AHPs before enzymatic hydrolysis under the applied chromatographic conditions. AHPs were enzymatically treated with β-mannosidase (B), β-glucosidase (C), pectinase (D), cellulase (E), and α-amylase (F). After digestion, detectable sugar fragment bands were observed following treatment with β-mannosidase, cellulase, and α-amylase, whereas no obvious bands appeared following treatment with β-glucosidase or pectinase. The different HPTLC profiles obtained after enzyme treatment suggest that AHPs contain carbohydrate structures with different susceptibilities to enzymatic hydrolysis. However, the specific glycosidic linkages cannot be identified from HPTLC results alone [33]. Cellulase and β-mannosidase treatments produced distinct HPTLC profiles, potentially reflecting differences in the hydrolysis products generated. However, the relatively weak bands observed following β-mannosidase and α-amylase treatment may reflect differences in the amount or chromatographic behavior of the resulting products [17]. The enzyme-assisted profiles are comparative hydrolysis evidence and do not establish a specific enzyme-binding site or cleavage linkage. Nevertheless, the specific glycosidic linkages in AHPs cannot be definitively identified based on HPTLC results alone, and further structural characterization is needed to confirm their identities.

### 3.6. Antioxidant Activity of AHPs from Different Origins

**Figure 3:**
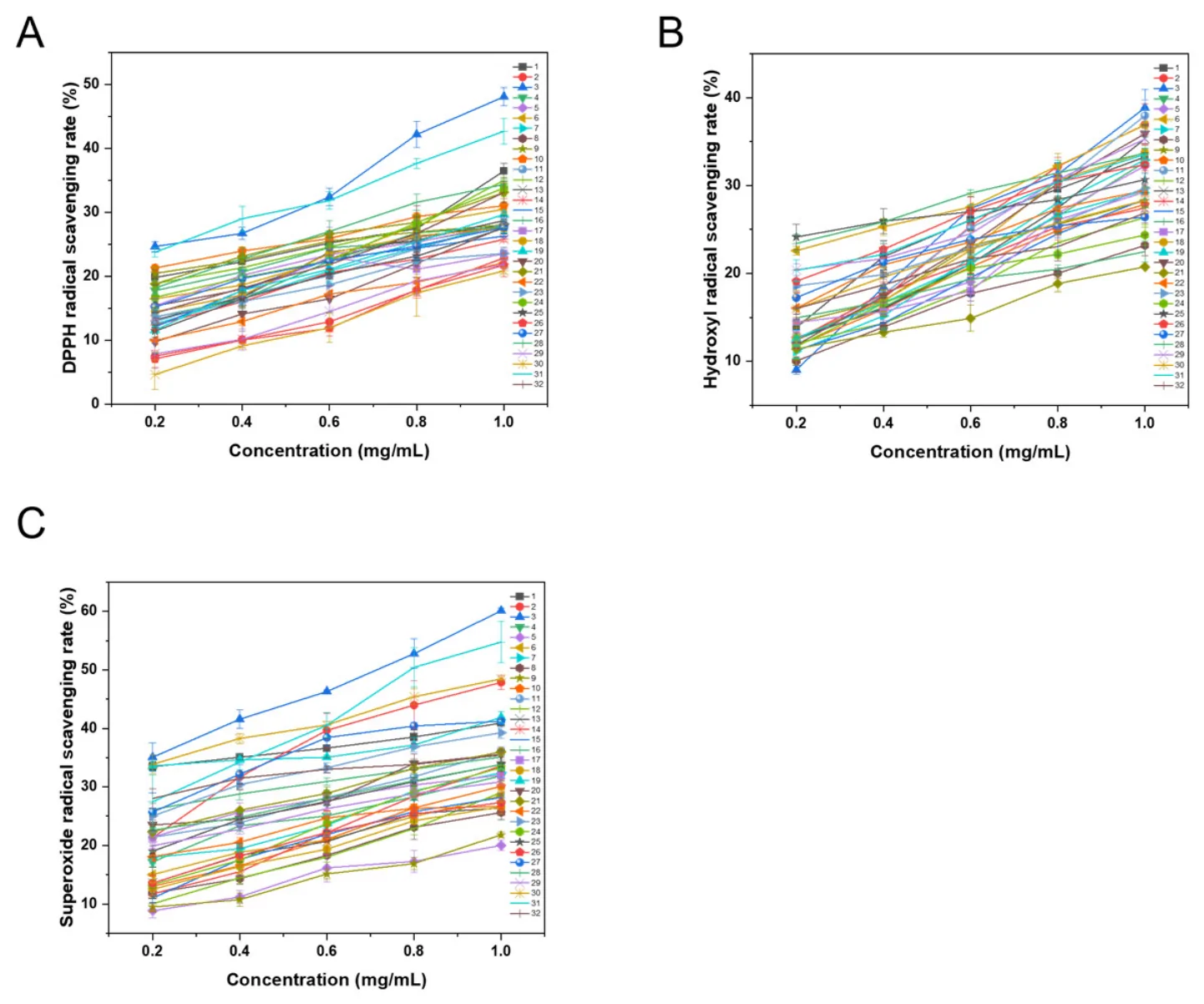
The antioxidant activity of AHPs from different origins in vitro: (**A**) DPPH, (**B**) •OH, (**C**) and •O_2_^−^.

Polysaccharides exhibit significant antioxidant capacity and have become popular in functional food development owing to their natural origin [34]. The influence of origin on the antioxidant capacity of AHPs has not yet been examined. This study examined the antioxidant capabilities of 32 AHPs derived from various sources. Figure 3A–C demonstrated the in vitro antioxidant ability of AHPs. All samples demonstrated the ability to scavenge DPPH, hydroxyl, and superoxide anion radicals, exhibiting concentration-dependent activity, hence affirming the stable antioxidant potential of AHPs, in accordance with Ma et al. [35]. Sample 3 exhibited higher radical scavenging activity than the other samples, indicating the strongest antioxidant potential among all tested AHPs.

**Figure 4:**
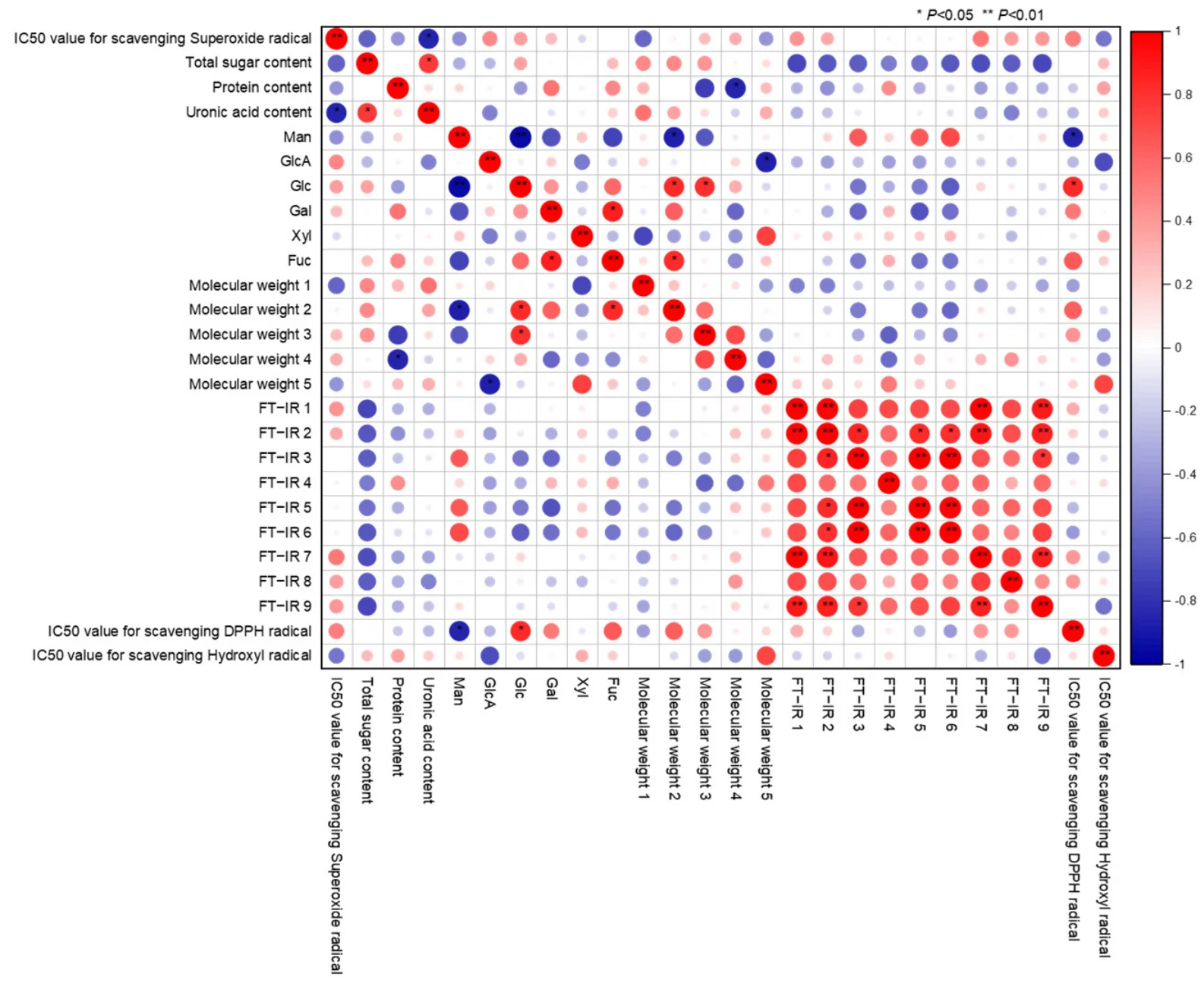
Pearson’s correlation analysis between the physicochemical properties and antioxidant activities of AHPs from different origins in vitro.

The antioxidant efficacy of polysaccharides correlates with their physicochemical characteristics [36]. The differences in total sugar, protein, uronic acid content (expressed as galacturonic acid equivalents) levels, monosaccharide composition, molecular weight, and infrared spectrum of AHPs may influence their antioxidant activity. Therefore, Pearson’s correlation analysis was performed on the antioxidant capacity and physicochemical properties of different AHP samples. As shown in Figure 4, the Glc content was significantly positively correlated with the DPPH free radical scavenging activity of AHPs (*p* < 0.05); that is, the increase in Glc content enhanced its scavenging ability, whereas the opposite was true for Man (*p* < 0.05). The IC_50_ value of superoxide anion radical scavenging capacity exhibited a strong negative correlation with the uronic acid content (expressed as galacturonic acid equivalents), suggesting that the uronic acid content (expressed as galacturonic acid equivalents) may be the primary determinant influencing the superoxide anion radical scavenging activity of AHPs. The scavenging ability of hydroxyl radicals was not substantially associated with the majority of the aforementioned physical and chemical indices, suggesting that it may depend on unobserved structural properties, such as the degree of branching and modifications of functional groups. The antioxidant activity of the AHPs exhibited considerable heterogeneity across various samples.

### 3.7. Lipid-Lowering and Hypoglycemic Potentials of AHPs from Different Origins

**Figure 5:**
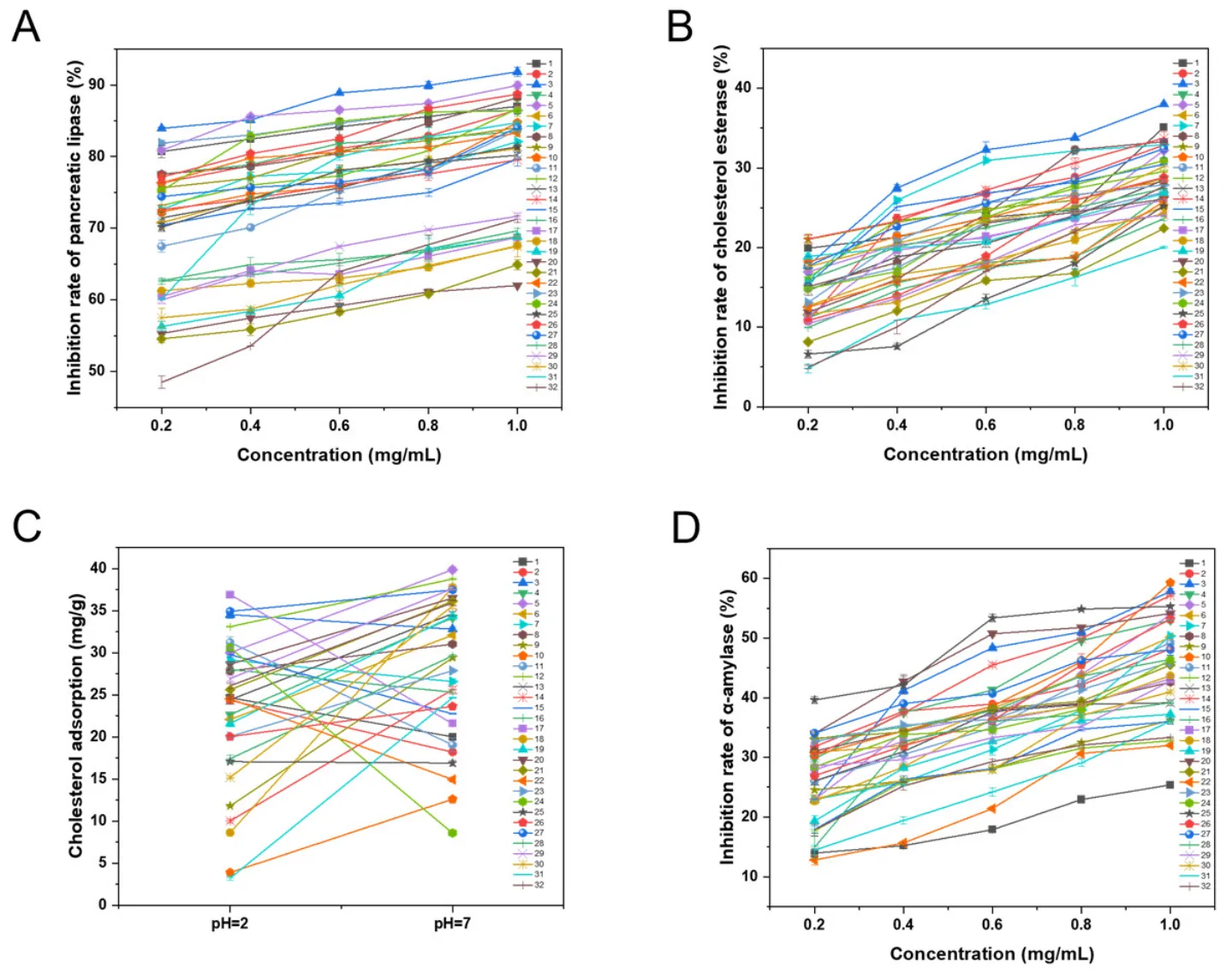
The lipid-lowering and hypoglycemic potentials of AHPs from different origins in vitro: (**A**) inhibition rate of pancreatic lipase, (**B**) the inhibition rate of cholesterol esterase, (**C**) the ability of cholesterol to adsorb, and (**D**) the inhibition rate of α -amylase.

Imbalanced or excessive dietary nutrition directly contributes to conditions such as obesity, elevated blood cholesterol, and type 2 diabetes [19,37,38]. Research has demonstrated that fungal polysaccharides can reduce blood glucose levels and improve dyslipidemia by suppressing cholesterol production [39,40]. To elucidate the influence of origin on the lipid-lowering and hypoglycemic properties of AHPs, 32 AHPs from various origins were examined for their efficacy in inhibiting pancreatic lipase, cholesterol esterase, and α-amylase, and their capacity to adsorb cholesterol in the gastric and intestinal milieu. Figure 5A–D showed the in vitro activity detection findings of AHPs from various origins, clearly demonstrating that all AHPs numbered 1–32 may block pancreatic lipase, cholesterol esterase, and α-amylase, while also exhibiting a notable cholesterol adsorption capability. As the concentration of AHPs rose, the inhibition rate and cholesterol adsorption of the three enzymes progressively increased, reflecting the typical features of concentration-dependent action. These results indicated that AHPs from different sources exhibited varying degrees of in vitro lipid-lowering and hypoglycemic potential, as evaluated by pancreatic lipase inhibition, cholesterol esterase inhibition, cholesterol adsorption, and α-amylase inhibition. Pancreatic lipase inhibition was assessed to investigate the potential of AHPs to interfere with dietary lipid digestion. However, this in vitro assay alone cannot establish whether AHPs can reduce blood lipid levels, and further in vivo studies are needed to verify their potential lipid-lowering effects.

**Figure 6:**
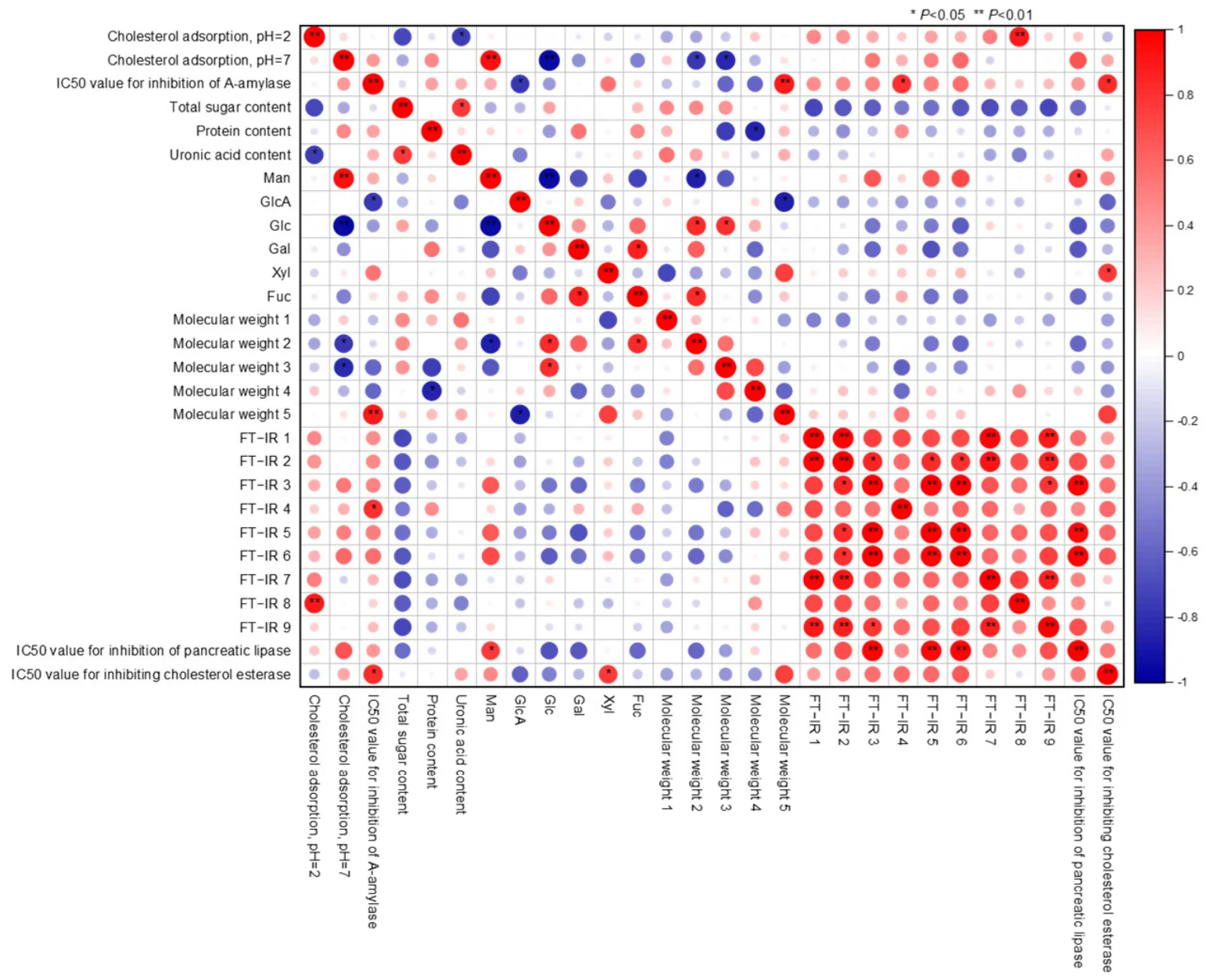
Pearson’s correlation analysis between the physicochemical properties and lipid-lowering and hypoglycemic potentials of AHPs from different origins in vitro.

The biological activity of natural polysaccharides is intricately linked to their physicochemical characteristics, with variations in origin often influencing their function indirectly through the regulation of their structure and composition [41]. Research indicated that the physicochemical features of AHPs differ by manufacturing region, encompassing sugar, protein, uronic acids (expressed as galacturonic acid equivalents), monosaccharides, and molecular weight. Pearson’s correlation analysis was employed to investigate the links between the activities of AHPs and their attributes (Figure 6). Man was positively correlated with the IC_50_ value for pancreatic lipase inhibition (*p* < 0.05), indicating that a higher Man content was associated with weaker inhibition under the assay conditions. FT-IR peaks at 1731, 1376, and 1245 cm^−1^ exhibited a strong correlation with lipase inhibition (*p* < 0.01). Xyl showed a favorable correlation with cholesterol esterase inhibition (*p* < 0.05). The uronic acid content (expressed as galacturonic acid equivalents) had a negative correlation with cholesterol adsorption at pH 2.0 (*p* < 0.05), whereas Glc demonstrated a negative correlation at pH 7 (*p* < 0.01). Peaks 2 and 3 were negatively correlated with adsorption (*p* < 0.05). Peak 5 and the FT-IR peak at 1656 cm^−1^ exhibited a positive correlation with α-amylase inhibition (*p* < 0.01, *p* < 0.05), whereas GlcA had a negative correlation (*p* < 0.05). Monosaccharides, molecular weight constituents, and FT-IR peaks were correlated with lipid-lowering effects, elucidating the mechanisms of AHP.

## 4. Discussion

The structural characteristics and biological activities of 32 AHP samples from different sources were investigated in this study. Although the samples shared similar major structural features, their chemical composition, monosaccharide proportions, and molecular weight distributions varied. These differences may contribute to variations in biological activities. Similar source-dependent structural variations have also been reported in polysaccharides isolated from different geographical origins of edible fungi [7], indicating that polysaccharide composition and structural characteristics may vary among sources and potentially be associated with differences in biological activities.

The uronic acid content varied among the AHP samples, indicating differences in their acidic characteristics. Uronic acids may influence polysaccharide physicochemical properties and intermolecular interactions [42]. However, their contents were not consistently associated with all functional activity indicators in this study, suggesting that their effects may depend on other structural factors. Although the AHP samples contained similar monosaccharide types, their Man and Glc proportions differed significantly, and correlation analysis revealed associations between these monosaccharides and antioxidant activity. In comparison, Su and Li [43] reported positive correlations between molecular weight and several radical scavenging activities, whereas fucose and galactose contents were negatively correlated with total antioxidant capacity. In the present study, antioxidant activity was associated with different structural parameters, including the proportions of mannose and glucose. These differences suggest that the structural characteristics associated with antioxidant activity may vary among fungal polysaccharides, depending on their composition and the antioxidant activity parameters evaluated. Nevertheless, correlation analysis alone cannot establish causality, and further structural characterization is required to clarify these relationships.

The AHPs from different sources all exhibited multi-component molecular weight distributions, but the proportions of each molecular weight component varied. Molecular weight differences may influence polysaccharide chain conformation, solubility, and intermolecular interactions [44]. In this study, associations between specific molecular weight components and functional activity suggest that molecular weight distribution, rather than molecular weight alone, may be relevant to the observed activity differences. However, the contributions of individual molecular weight fractions remain to be determined. The FT-IR results showed that the AHPs from different sources have similar functional group characteristics, suggesting that their core structure is relatively conserved; however, the differences in absorption intensity indicate changes in the local structural environment. Similarities in FT-IR profiles indicate comparable major chemical features but do not necessarily imply identical polysaccharide structures. The HPTLC analysis further showed that AHPs from different sources shared similar detectable carbohydrate profiles, although differences in their chromatographic patterns suggested variations in carbohydrate composition or relative abundance. However, HPTLC alone cannot definitively identify specific glycosidic linkages. Further analyses, such as methylation analysis and nuclear magnetic resonance spectroscopy, are needed to clarify these structural differences.

Different sources of AHPs exhibited varying antioxidant activities, which may be associated with differences in their structural characteristics. In the present study, correlation analysis revealed associations between antioxidant activity and several parameters, including monosaccharide composition and uronic acid content (expressed as galacturonic acid equivalents). Furthermore, previous studies on *Auricularia* polysaccharides have reported associations between molecular weight and antioxidant activity [43], while carboxymethylation has been shown to enhance the in vitro antioxidant activity of *Auricularia* polysaccharides [45]. These findings suggest that both intrinsic structural characteristics and chemical modification may be associated with polysaccharide antioxidant activity. Although residual proteins may contribute to antioxidant activity, protein content did not show a consistent association with antioxidant capacity among the AHP samples, indicating that protein content alone is unlikely to explain the observed differences. However, potential contributions from residual proteins and other non-polysaccharide components cannot be completely excluded. Differences in sample purity, extraction procedures, and antioxidant assay conditions should also be considered when comparing results across studies. Further investigations using purified polysaccharide fractions and detailed structural characterization are needed to clarify the contributions of individual structural features to antioxidant activity.

Additionally, different sources of AHPs exhibited varying lipid-related activities and enzyme inhibitory potential associated with carbohydrate metabolism. Correlation analysis showed that Man, Xyl, uronic acids (expressed as galacturonic acid equivalents), and some FT-IR characteristic peaks were associated with related activity indicators, suggesting that the composition of monosaccharides and functional group characteristics may be related to these functional properties. Comparable in vitro lipid-related effects have been reported for soluble dietary fiber from *Hericium erinaceus*, which exhibited pancreatic lipase inhibition and cholesterol micelle adsorption [19]. Although differences in material composition and experimental conditions limit direct comparisons, these findings provide a relevant reference for the lipid-related activities observed in the present study. Pancreatic lipase inhibition may be beneficial for selected individuals, such as those with obesity, but may not be advantageous for everyone. Excessive or prolonged inhibition may impair dietary fat digestion and the absorption of fat-soluble vitamins, potentially increasing the risk of nutritional deficiencies. Therefore, the potential application of AHPs for lipid management should be evaluated in terms of both efficacy and safety. The correlations identified in this study do not establish direct interactions between AHPs and target enzymes or demonstrate causal relationships between specific structural features and functional activities. Moreover, the present in vitro results do not establish that AHPs reduce blood lipid or glucose levels in vivo; further animal studies and mechanistic investigations are needed to evaluate these potential effects.

## 5. Conclusions

In this study, 32 AHPs from different geographical origins were systematically characterized and evaluated for their structural properties and biological activities. The results showed that AHPs were mainly composed of Man, Glc, GlcA, Gal, Xyl, and Fuc, with molecular weights ranging from 3.6 to 6820 kDa, and exhibited varying degrees of antioxidant activity and in vitro enzyme inhibitory and lipid-related activities. Correlation analysis revealed that monosaccharide composition, particularly Glc, Man, and uronic acid content (expressed as galacturonic acid equivalents), was associated with antioxidant activity. In addition, Xyl content, specific molecular weight peaks (peaks 2, 3 and 5), and characteristic FT-IR bands (1731, 1376, 1245, and 1656 cm^−1^) showed correlations with enzyme inhibitory activities related to glucose and lipid metabolism. This study provides a theoretical basis for the quality evaluation and rational selection of AHPs as potential functional food ingredients. Further in vivo and mechanistic studies are needed to verify their potential benefits in glucose and lipid metabolism.

## Figures and Tables

**Table 1:** Information on *Auricularia heimuer* (*A. heimuer*) collected from different provinces and autonomous regions of China.

Sample Number	City/County	
1	Heilongjiang	Yichun
2	Heilongjiang	Mudanjiang
3	Shaanxi	Zhashui
4	Yunnan	Kunming
5	Jilin	Wangqing, Yanbian
6	Jilin	Heishan, Panshi
7	Tibet	Linzhi
8	Sichuan	Chengdu
9	Heilongjiang	Hailin
10	Heilongjiang	Dongning
11	Jilin	Antu, Yanbian
12	Hubei	Suizhou
13	Jilin	Dunhua
14	Heilongjiang	Jixi
15	Inner Mongolia	Hulunbuir
16	Henan	Zhumadian
17	Fujian	Sanming
18	Henan	Zhoukou
19	Henan	Sanmenxia
20	Jiangxi	Jinggangshan
21	Fujian	Wuyishan
22	Jiangxi	Yichun
23	Yunnan	Honghe Hani Yi Autonomous Prefecture
24	Xinjiang	Aksu
25	Shandong	Linyi
26	Hubei	Shiyan
27	Gansu	Longnan
28	Anhui	Huangshan
29	Zhejiang	Lishui
30	Jilin	Quchaihe, Panshi
31	Jilin	Huangsongdian, Jilin
32	Jiangxi	Jinxian

**Table 2:** Determination of content in AHPs.

Sample Number	Total Sugar Content (%)	Protein Content (%)	Uronic Acid Content (Expressed as Galacturonic Acid Equivalents, %)
1	64.78 ± 0.11 ^e^	2.33 ± 0.60 ^e^	16.11 ± 0.14 ^jk^
2	73.00 ± 1.10 ^j^	4.14 ± 0.40 ^g^	16.59 ± 0.43 ^kL^
3	79.90 ± 0.55 ^L^	1.39 ± 0.06 ^bcd^	13.37 ± 0.10 ^de^
4	65.11 ± 0.88 ^ef^	1.73 ± 0.40 ^cde^	19.42 ± 0.38 ^n^
5	68.62 ± 0.88 ^gh^	4.25 ± 1.51 ^g^	13.37 ± 0.48 ^de^
6	57.99 ± 0.55 ^ab^	4.14 ± 0.40 ^g^	12.31 ± 0.10 ^b^
7	65.22 ± 0.11 ^ef^	4.75 ± 0.20 ^gh^	20.34 ± 0.43 ^o^
8	71.25 ± 0.44 ^i^	3.14 ± 0.20 ^f^	14.47 ± 0.34 ^fg^
9	75.41 ± 0.44 ^k^	1.73 ± 0.40 ^cde^	15.38 ± 0.29 ^hij^
10	68.73 ± 0.99 ^gh^	0.93 ± 0.40 ^abc^	12.79 ± 0.29 ^bcd^
11	58.65 ± 1.21 ^b^	2.13 ± 0.40 ^de^	12.31 ± 0.10 ^b^
12	58.54 ± 1.31 ^b^	5.55 ± 0.60 ^ij^	13.94 ± 0.67 ^ef^
13	58.10 ± 0.66 ^ab^	5.96 ± 0.60 ^j^	12.21 ± 0.10 ^b^
14	73.11 ± 0.99 ^j^	2.33 ± 0.20 ^e^	16.01 ± 0.63 ^jk^
15	73.44 ± 1.10 ^j^	4.14 ± 0.40 ^g^	17.02 ± 0.10 ^L^
16	56.89 ± 0.33 ^a^	7.16 ± 0.20 ^k^	10.72 ± 0.43 ^a^
17	58.86 ± 0.11 ^b^	0.72 ± 0.20 ^ab^	12.50 ± 0.29 ^bc^
18	63.03 ± 1.21 ^d^	1.53 ± 0.20 ^bcde^	16.54 ± 0.48 ^kL^
19	62.48 ± 0.66 ^d^	1.73 ± 0.40 ^cde^	15.87 ± 0.48 ^ijk^
20	58.10 ± 1.10 ^ab^	0.72 ± 0.20 ^ab^	14.86 ± 0.24 ^gh^
21	58.97 ± 0.44 ^b^	2.33 ± 0.20 ^e^	12.88 ± 0.19 ^bcd^
22	69.93 ± 0.22 ^h^	1.73 ± 0.40 ^cde^	13.17 ± 0.38 ^bcd^
23	67.52 ± 0.22 ^g^	9.18 ± 0.20 ^L^	15.24 ± 0.34 ^hi^
24	71.58 ± 0.77 ^i^	4.14 ± 0.40 ^g^	12.65 ± 0.52 ^bcd^
25	71.25 ± 0.88 ^i^	4.55 ± 0.40 ^gh^	15.96 ± 0.58 ^jk^
26	66.32 ± 0.11 ^f^	0.93 ± 0.40 ^abc^	18.56 ± 0.48 ^m^
27	64.56 ± 0.11 ^e^	5.35 ± 0.40 ^hij^	13.94 ± 0.10 ^ef^
28	60.29 ± 0.66 ^c^	0.20 ± 0.08 ^a^	12.31 ± 0.58 ^b^
29	69.17 ± 0.77 ^h^	4.14 ± 0.40 ^g^	16.35 ± 0.67 ^kL^
30	63.25 ± 0.55 ^d^	4.55 ± 0.40 ^gh^	16.11 ± 0.43 ^jk^
31	69.17 ± 0.55 ^h^	1.93 ± 0.20 ^de^	16.92 ± 0.10 ^L^
32	62.81 ± 0.55 ^d^	4.95 ± 0.40 ^gh^	19.38 ± 0.43 ^n^

Notes: The values were expressed as means ± SD (*n* = 3). The mean values followed by different superscripts in the same column were significantly different (*p* < 0.05).

**Table 3:** Monosaccharide composition ratio of AHPs.

Sample Number	Monosaccharide Composition (mol%)					
	Man	GlcA	Glc	Gal	Xyl	Fuc
1	53.7	8.6	16.8	10.7	7.2	2.9
2	53.3	9.8	13.8	11.7	8.1	3.2
3	47.4	5.5	20.5	16.1	5.3	5.2
4	55.9	9.2	13.8	10.8	6.9	3.4
5	45.8	8.4	26.1	11.0	5.9	2.7
6	40.7	7.4	28.4	16.3	3.2	4.0
7	43.9	8.5	18.2	18.7	5.2	5.5
8	41.2	7.1	36.2	7.3	5.6	2.6
9	32.3	7.1	27.8	16.7	11.4	4.7
10	35.0	7.5	29.6	17.8	5.9	4.1
11	53.0	9.8	23.6	7.9	5.7	--
12	54.7	7.8	24.4	8.4	2.6	2.2
13	50.4	8.8	22.8	10.8	5.2	1.9
14	40.5	7.5	37.3	8.5	4.1	2.2
15	45.3	8.7	25.4	11.7	5.2	3.6
16	50.4	2.5	18.7	15.5	7.5	5.4
17	33.1	6.8	42.6	10.3	4.6	2.7
18	54.3	9.6	19.5	9.4	7.2	--
19	58.1	5.8	22.0	5.4	8.6	--
20	58.6	8.8	20.3	5.8	6.5	--
21	65.6	4.5	16.8	7.3	5.8	--
22	62.1	6.6	8.8	12.0	6.8	3.7
23	33.7	6.8	36.8	14.4	4.2	4.1
24	61.1	5.6	17.7	9.3	6.3	--
25	57.8	5.4	24.1	7.3	5.4	--
26	53.0	6.2	21.7	10.0	6.8	2.3
27	54.0	6.0	24.5	7.8	5.7	2.0
28	63.9	5.5	20.2	3.6	6.7	--
29	64.0	6.8	15.4	6.5	7.4	--
30	54.8	5.8	22.8	8.2	6.3	2.1
31	64.8	6.7	13.8	7.9	6.8	--
32	65.1	7.3	12.1	7.4	8.2	--

**Table 4:** Molecular weights corresponding to five chromatographic peaks of AHPs from different regions.

Sample Number	Molecular Weight (kDa)				
	Peak 1 (kDa)	Peak 2 (kDa)	Peak 3 (kDa)	Peak 4 (kDa)	Peak 5 (kDa)
1	6548.7	3391.6	11.3	--	--
2	6820	486.6	12.7	--	--
3	5932.5	755.2	12.4	3.6	--
4	6747.2	890.4	12.4	--	--
5	4928.8	2792	1219.9	12	--
6	3715.3	12.4	--	--	--
7	5457.4	3619.8	1690.6	13.1	--
8	3738.1	12.6	4.1	--	--
9	4947.7	1245.4	11.8	4.0	--
10	3630.9	2247.9	12.7	--	--
11	2966.2	1715.4	11.8	3.8	--
12	6553.7	3030.5	1601.1	12.6	3.6
13	5184.4	2636.1	1407.8	11.5	3.8
14	6351.1	3687.0	2850.4	12.9	3.7
15	5485.1	1067.7	13.5	3.6	--
16	5928.0	3633.7	12.9	4.1	--
17	4906.2	3675.7	2614.0	12.9	4.1
18	5923.5	2602.0	1396.0	11.8	4.1
19	6042.7	3428.2	1529.2	12.7	4.1
20	6454.0	4791.1	1091.7	12.8	4.4
21	4943.9	1203.2	11.6	4.5	--
22	6716.3	754.7	12.4	4.7	--
23	6005.7	4193.3	1656.0	10.7	4.5
24	2749.6	12.8	4.5	--	--
25	5571.4	1011.2	11.2	4.6	--
26	3279.2	12.2	4.6	--	--
27	5512.0	2504.3	1437.2	11.8	4.7
28	3944.0	2200.2	13.2	--	--
29	5855.8	3396.8	1767.4	11.8	4.4
30	5424.0	3063.1	1581.6	11.5	4.8
31	418	15.8	7.7	--	--
32	401	16.9	7.4	--	--

**Table 5:** Enzyme-assisted HPTLC analysis of AHPs.

Sample Number	HPTLC Band Detection				
	β-Mannosidase	β-Glucosidase	Pectinase	Cellulase	α-Amylase
1	+	−	−	+	+
2	+	−	−	+	+
3	+	−	−	+	+
4	+	−	−	+	+
5	+	−	−	+	+
6	+	−	−	+	+
7	+	−	−	+	+
8	+	−	−	+	+
9	+	−	−	+	+
10	+	−	−	+	+
11	+	−	−	+	+
12	+	−	−	+	+
13	+	−	−	+	+
14	+	−	−	+	+
15	+	−	−	+	+
16	+	−	−	+	+
17	+	−	−	+	+
18	+	−	−	+	+
19	+	−	−	+	+
20	+	−	−	+	+
21	+	−	−	+	+
22	+	−	−	+	+
23	+	−	−	+	+
24	+	−	−	+	+
25	+	−	−	+	+
26	+	−	−	+	+
27	+	−	−	+	+
28	+	−	−	+	+
29	+	−	−	+	+
30	+	−	−	+	+
31	+	−	−	+	+
32	+	−	−	+	+

Notes: “+” indicates that detectable bands were observed following enzymatic treatment, whereas “−” indicates that no obvious bands were detected under the applied HPTLC conditions. These results reflect the observed chromatographic profiles and do not independently establish the presence or absence of specific glycosidic linkages.

## Data Availability

The original data are available from the corresponding authors on reasonable request.

## Supplementary Materials

The following supporting information can be downloaded at: https://www.mdpi.com/article/10.3390/foods15213816/s1, Figure S1: HPTLC of AHPs by different enzymatic hydrolysis methods.
