# Supplementary material for: The Saccharide Mapping and Biological Activity Analysis of Auricularia heimuer Polysaccharides from Different Origins In Vitro
Source: Foods. 2026 Oct 26;15(21):3816. doi: 10.3390/foods15213816 (PMC13762665; doi:10.3390/foods15213816)
Supplement: Supplementary file 1 [file foods-15-03816-s001.zip › foods-4598212-supplementary.pdf]

Supplementary data

The saccharide mapping and biological activity analysis of *Auricularia heimuer* polysaccharides from different origins in vitro

Miao Ding [1], Zixuan Wang [1], Hongxiang Liu [1], Yingdi Dai [1], Yang Liu [1,*] and Qi Wang [1,*]

[1] Engineering Research Center of Chinese Ministry of Education for Edible and Medicinal Fungi, College of Plant Protection, College of Mycology, Jilin Agricultural University, Changchun 130118, China; miaoding@mails.jlau.edu.cn (M.D.); 13948530370@163.com (Z.W.); hongxiangliu@jlau.edu.cn (H.L.); daiyingdi@mails.jlau.edu.cn (Y.D.)

* Correspondence: y_liu10@jlau.edu.cn (Y.L.); qiwang@jlau.edu.cn (Q.W.)

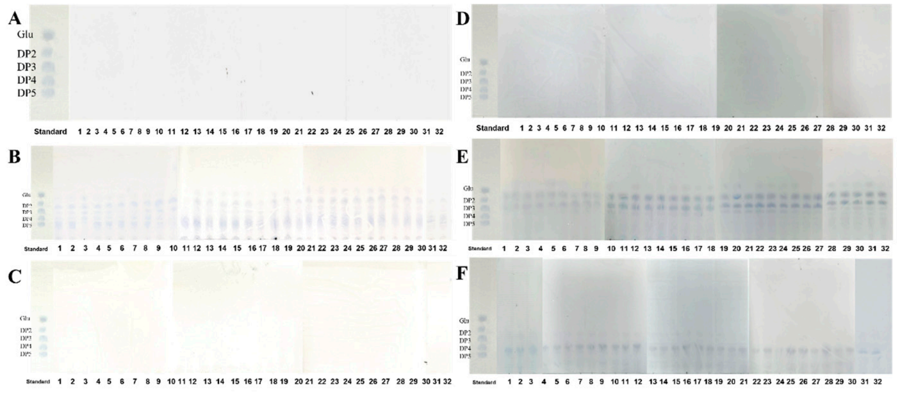

Figure S1. HPTLC of AHPs by different enzymatic hydrolysis methods

A: Before enzymatic hydrolysis, B: β-Mannosidase, C: β-Glucosidase, D: Pectinase, E: Cellulase, F: α-Amylase (DP2: maltose, DP3: maltotriose, DP4: maltotetraose, DP5: maltpentaose)
